# Manipulation of neuronal activity by an artificial spiking neural network implemented on a closed-loop brain-computer interface in non-human primates

**DOI:** 10.1088/1741-2552/adec1c

**Published:** 2025-07-21

**Authors:** Jonathan Mishler, Richy Yun, Steve Perlmutter, Rajesh P N Rao, Eberhard Fetz

**Affiliations:** 1 Department of Bioengineering, University of Washington, Seattle, WA, United States of America; 2 Washington National Primate Research Center, University of Washington, Seattle, WA, United States of America; 3 Paul G. Allen School of Computer Science and Engineering, University of Washington, Seattle, WA, United States of America; 4 Department of Neurobiology & Biophysics, University of Washington, Seattle, WA, United States of America; 5 Center for Neurotechnology, University of Washington, Seattle, WA, United States of America

**Keywords:** spiking neural networks, closed-loop, intracortical microstimulation, brain-computer interface

## Abstract

*Objective.* Closed-loop brain-computer interfaces can be used to bridge, modulate, or repair damaged connections within the brain to restore functional deficits. Towards this goal, we demonstrate that small artificial spiking neural networks can be bidirectionally interfaced with single neurons (SNs) in the neocortex of non-human primates (NHPs) to create artificial connections between the SNs to manipulate their activity in predictable ways. *Approach.* Spikes from a small group of SNs were recorded from primary motor cortex of two awake NHPs during rest. The SNs were then interfaced with a small network of integrate-and-fire units (IFUs) that were programmed on a custom clBCI. Spikes from the SNs evoked excitatory and/or inhibitory postsynaptic potentials in the IFUs, which themselves spiked when their membrane potentials exceeded a predetermined threshold. Spikes from the IFUs triggered single pulses of intracortical microstimulation (ICMS) to modulate the activity of the cortical SNs. *Main results.* We show that the altered closed-loop dynamics within the cortex depends on several factors including the connectivity between the SNs and IFUs, as well as the precise timing of the ICMS. We additionally show that the closed-loop dynamics can reliably be modeled from open-loop measurements. *Significance.* Our results demonstrate a new type of hybrid biological-artificial neural system based on a clBCI that interfaces SNs in the brain with artificial IFUs to modulate biological activity in the brain. Our model of the closed-loop dynamics may be leveraged in the future to develop training algorithms that shape the closed-loop dynamics of networks in the brain to correct aberrant neural activity and rehabilitate damaged neural circuits.

## 1. Introduction

The mammalian neocortex utilizes interconnected neural networks to integrate and process multimodal sensory information to perform complex tasks. Several diseases such as stroke and depression disrupt function in these networks and are accompanied by behavioral deficits. For instance, stroke disrupts communication across neural networks by lesioning focal regions and severing long-range connections and impairing information processing [1]. The limited treatment options for these diseases necessitate the development of new tools to rehabilitate the brain. One set of approaches investigate how bidirectional closed-loop brain-computer interfaces (clBCIs) can serve as neuroprostheses by simultaneously decoding information from—and stimulating the brain to restore lost function by inducing neuroplasticity [2–4] or by chronically modulating activity within pathological circuits to produce a clinical benefit [5–7].

For instance, in humans subjects, some studies have implemented closed-loop EEG paradigms that deliver transcranial magnetic or electric stimuli with the goal of treating various neurological disorders [3, 4, 8]. Closed-loop devices are also being developed for deep-brain stimulation paradigms with the goal of modulating aberrant neural activity only when necessary [5, 6, 9]. In animals, studies have explored how clBCIs can be used to treat various disorders such as epilepsy [10, 11], pain [11, 12], traumatic brain injury [13], and fear dysregulation [14]. Beyond experimental work, computational efforts have also explored how machine learning can be utilized to restore lost motor function in lesioned cortical models [15, 16]. Such devices can be thought of as ‘neural co-processors’ that leverage machine learning to co-learn and co-adapt with the brain to optimize the closed-loop stimulation [16–18]. clBCIs that interface with collections of single neurons (SNs) *in vivo*, which would enhance their ability to interact with local circuits more precisely, have been less explored. In our previous work, we found that the precise timing of intracortical microstimulation (ICMS) relative to a SN’s previous spike time had a significant effect on its evoked-spike probability, and thus the ratio of the evoked excitation to inhibition [19]. One limitation in conventional clBCI applications is that despite leveraging closed-loop control, they typically ignore this intrinsic temporal dependency and apply stimulation at fixed or behaviorally-triggered latencies. Incorporating neuron-specific timing rules into stimulation protocols could significantly enhance clBCI effectiveness by aligning stimulation with periods of heightened excitability, thereby increasing the likelihood of evoking spikes to optimally shape network activity and/or drive synaptic plasticity. Therefore, the clBCI should be able to simultaneously decode spike activity from a collection of SNs while precisely timing the ICMS to control the evoked response. Furthermore, the clBCI should also be capable of continuously adjusting its parameters to co-learn and co-adapt with the brain to account for non-stationarities in neural activity. Given these requirements, SNNs are attractive candidates to serve as the computational substrate for a clBCI. Unlike traditional rate-based models, SNNs inherently operate in the time domain and can exploit the precise timing of spikes for both decoding and stimulation control, making them well-suited for modulating neural circuits where spike timing governs excitability and plasticity. Additionally, their architecture supports biologically inspired learning rules such as STDP [20], enabling real-time adaptation and closed-loop co-plasticity. SNNs can also be implemented on low-power neuromorphic hardware, which is advantageous for chronic implants due to reduced heat, latency, and power constraints [21, 22].

The primary goal of this work was to demonstrate that artificial SNNs, when used to trigger single pulses of ICMS, can be interfaced with cortical SNs to establish artificial connections within cortical networks and modulate their intrinsic spiking activity. A secondary objective was to develop a computational model that accurately captured the closed-loop dynamics of this system, laying the groundwork for future training algorithms enabling context-dependent control of neural activity. To this end, we programmed a small artificial neural network of integrate-and-fire units (IFUs) on a custom clBCI [23] and interfaced this artificial network with biological SNs in awake NHPs. The SNs evoked artificial excitatory or inhibitory postsynaptic potentials (‘EPSPs’ or ‘IPSPs’) in the IFUs to excite or inhibit them, respectively. When the IFUs ‘spiked’, they triggered a single pulse of ICMS to modulate the activity of one of the SNs, thereby creating artificial ‘connections’ between the SNs and IFUs. It is important to note that we did not train these hybrid biological and artificial neural networks to control the activity of the SNs, but rather first explored how both ICMS, and various features of the clBCI altered closed-loop activity. This is important since any successful training algorithm will first require a basic understanding of how the closed-loop dynamics operate experimentally. Based on these pilot experiments, we created a model of the closed-loop dynamics of the artificial-biological neural network system that accurately predicted the changes in SN spike activity during stimulation. The model was then used to explore how features of the clBCI shape the closed-loop dynamics. Finally, we used the model to investigate an important limitation on the performance of multichannel clBCIs, namely, the effect of stimulus artifacts on accurate detection of SN spikes. We conclude by discussing how the model could be used in future work to train artificial neural networks of IFUs for clinically meaningful applications.

## 2. Materials and methods

### 2.1. Implants and surgery

**Figure 1.**
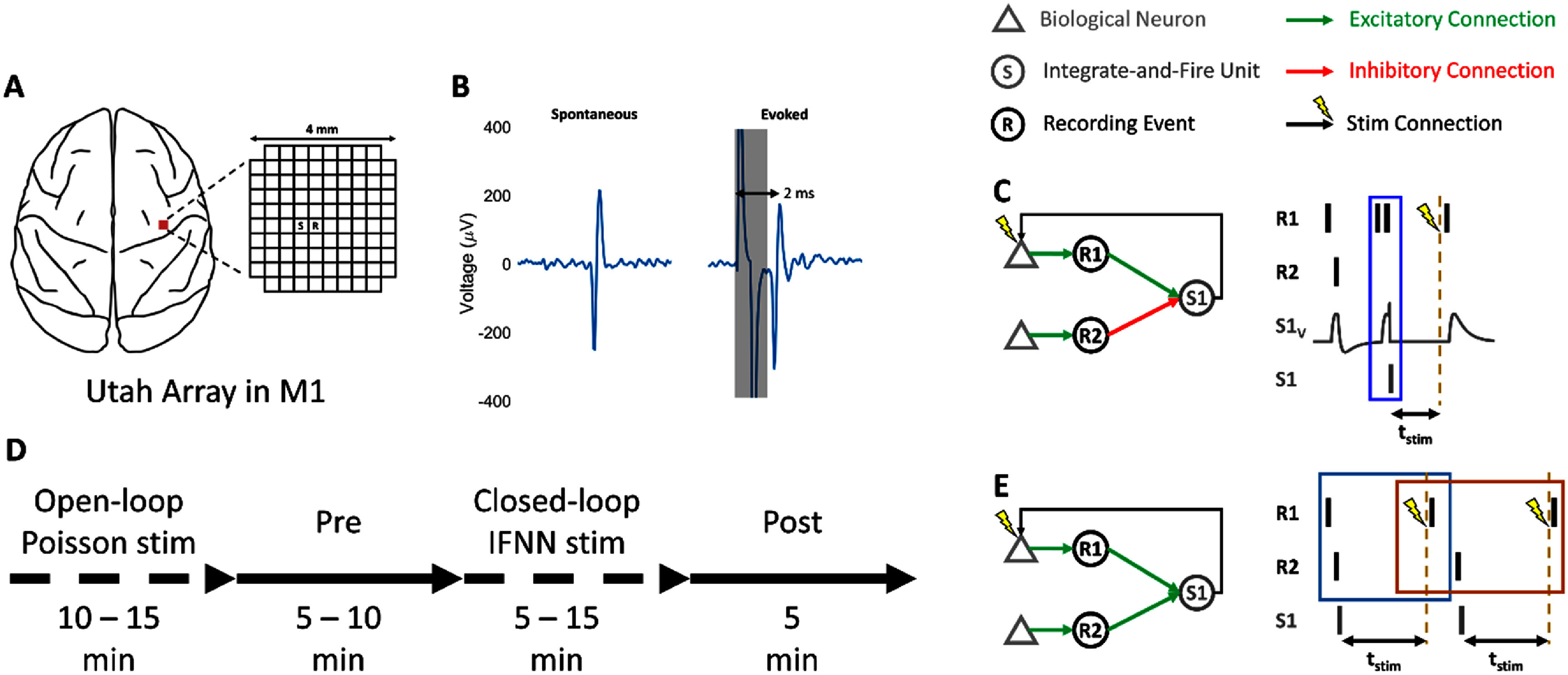
Experimental design. (A) Diagram showing placement of Utah array in primary motor cortex. The stimulation channels (S) were always adjacent to the recording channels (R), as shown in the example here, to reliably evoke spikes in the SNs. (B) Example of a spontaneous and evoked spike. Shaded area shows stimulation artifact, which is followed 2 ms later by an evoked spike. (C) Example showing how spikes recorded from two SNs (triangles in left panel) on Neurochip3 Recording Events (R1 and R2) cause PSPs in artificial neuron S1 (S1_v_ in right panel), which spikes when its membrane potential reaches a fixed threshold. When S1 spikes, it sends a trigger to stimulate one of the two SNs (in this example, the top SN in the left panel) after a delay *t*_stim_. (D) Typical experimental timeline, starting with open-loop Poisson distributed stimulation to build a library of SN responses to ICMS, followed by the recording of spontaneous SN activity (Pre). The SNs were then interfaced with the IFUs in an clBCI implemented on the clBCI during closed-loop stimulation. This was sometimes followed by a recording of spontaneous SN activity (Post) to measure any lasting effects from the ICMS. (E) Example of a feedback loop generated by the clBCI. Spontaneous spikes from the SNs can trigger stimulation via the clBCI to evoke new spikes in SNs (second spike in R1 in the right panel). The spontaneous spikes that trigger stimulation are grouped together with the evoked spikes to create an ‘elementary pattern’ (blue box and orange box). Elementary patterns with overlapping spikes are grouped together to identify the complete feedback loop and its size (the number of spikes in the loop). The size of the feedback loop in the example shown here is 5 spikes.

Two *Macaca Nemestrina* NHPs were chronically implanted unilaterally in the hand region of primary motor cortex with a single 96-channel Utah microelectrode array (figure 1(A)) (10 × 10 grid, 400 *µ*m inter-electrode spacing, 1.5 mm depth, iridium oxide, Blackrock Microsystems). Sterile surgeries were performed with isoflurane anesthesia under aseptic conditions and continuous monitoring of all vitals. A skin incision was first made to expose the skull. A 1.5 cm square craniotomy centered 4 mm over bregma was then performed to expose the dura, which was cut along three sides to expose the cortex. The arrays were then implanted, after which the dura was sutured around the implant. Two reference wires were placed between the cortex and the dura, and two were placed between the dura and the skull. The bone flap that was removed from the craniotomy was then reattached to the skull with a titanium strap. A second titanium strap was connected to the skull with screws to secure the wire bundle that connected the array to the recording pedestal, which itself was secured to the skull with screws. The skin was then sutured around the base of the pedestal. Following surgery, the animals received postoperative courses of antibiotics and analgesics. All procedures conformed to the National Institutes of Health *Guide for the Care and Use of Laboratory Animals* and were approved by the University of Washington Institutional Animal Care and Use Committee.

### 2.2. Electrophysiology

Neural activity was recorded on one of two systems—the Neurochip3 (32 channels, 20 kHz sampling rate) [23], or the neural interface processor (Ripple Neuro, 96 channels, 30 kHz sampling rate). Closed-loop experiments were exclusively performed on the Neurochip3. The Ripple system was occasionally used to record spontaneous activity from all SNs simultaneously to identify which SNs would be used that day. The raw voltage signals for each channel were first bandpass filtered to isolate spike activity. The low cutoff frequency ranged from 1000–2750 Hz (SN-specific), with a fixed high cutoff at 5000 Hz. This variable low cutoff was necessary to ensure reliable detection of both spontaneous and evoked spikes (figure 1(B)). SN spikes were then identified using two-window discrimination. Briefly, a voltage threshold was set for each SN to detect candidate spikes. Each detected waveform was required to pass through two sequential time-amplitude windows aligned with the negative peak and subsequent positive deflection of the spike waveform within a defined time interval. This method improves specificity by rejecting noise and waveforms that do not match the characteristic shape of the targeted SN’s action potential. More details of this spike discriminator can be found in [23]. Stimuli consisted of single biphasic pulses (cathodal first, 200 *µ*s phase widths, 15 *µ*A). The stimulation parameters were chosen to reliably evoke spikes in each SN (25%–95% evoked spike probability across all SNs) while maintaining focal activation and minimizing current spread. In each experiment, we utilized the Intan fast settle function to zero out the analog signal following stimulation for 400 *µ*s to prevent amplifier saturation and facilitate the detection of the evoked spikes.

### 2.3. Spiking neural network model

We programmed the IFUs with current-based synapses [24] on a Cyclone V FPGA on the Neurochip3 [23]. Each SN could have an excitatory, inhibitory, or no connection to each of the IFUs. The clBCI could support up to a combined total of eight SNs and IFUs. Spikes from the SNs were recorded via the Neurochip3 ‘Recording Events.’ For these initial pilot experiments, we did not adhere to Dale’s principle (i.e. we allowed connections from the same SN to be excitatory or inhibitory). The PSPs sent from an SN, *i*, to an IFU, *j*, were modeled as the difference between two exponential functions that were stored on the Neurochip3 and updated at a sampling rate of 10 kHz:

\begin{align*}{ }{S_{i,j{ }t + 1}} = {C_{{\text{Slow Decay}}}} \times {S_{i,j{ }t}}\end{align*}

\begin{align*}{F_{i,j{ }t + 1}} = {C_{{\text{Fast Decay}}}} \times {F_{i,j{ }t}}\end{align*}

\begin{align*}{\text{PS}}{{\text{P}}_{i,j{ }t + 1}} = {S_{i,j{ }t + 1}} - { }{F_{i,j{ }t + 1}}\end{align*}

where *S_i,j_* and *F_i,j_* are recursively defined exponential functions whose decay rates are defined by *CSlow Decay* and *CFast Decay*. We calibrated the values of *C*_Slow Decay_ and *C*_Fast Decay_ to achieve rise-time and decay-time constants of 1.5 and 4 ms, respectively. The strengths of the connections between each SN, *i*, and IFU, *j*, were stored in a weight matrix, **W**. Every time an SN, *i*, spiked, the values of both *S_i,j_* and *F_i,j_* were increased by *w_i,j_* following a conduction delay of 0.1 ms. An IFU spiked when its membrane potential reached a predefined threshold, *T_j_*, after which it was reset to 0 (figure 1(C)).

The thresholds, rise and decay time constants, and refractory periods were identical for each IFU. For our experiments, we set the refractory period to 0 ms. Each output IFU had a delay, *t*_stim_ between when it spiked and when stimulation was delivered. The values of *t*_stim_ for each IFU were chosen to be at least 4 ms apart from one another to enable the detection of the spikes evoked by stimulation with minimal interference from stimulation artifacts. The complete set of Neurochip3 parameters for each experiment will be published (link in Data Availability section) with this article to facilitate the reproducibility of this work.

### 2.4. Experimental design

The NHPs were trained to calmly sit in a primate chair while receiving a periodic smoothie to maintain their attention; no other task was performed during the study. Our experiments began by selecting the set of SNs, stimulation sites, weights, and stimulation delays between the spikes of each output IFU and the corresponding ICMS pulse (figure 1(C)). We tested different clBCI features to explore their effects on the closed-loop operation, including recurrent connections and hidden layers. We selected the sets of SNs for each experiment based on their signal-to-noise ratios, as well as the ability for a spike to be reliably evoked by ICMS on an adjacent channel. We manually calibrated the clBCI excitatory weights to be large enough such that any two spikes from the SNs that occurred within 1.1–12.6 ms of each other would cause the connected IFUs to spike (corresponding to EPSP amplitudes of 52.5%–97.5% of the IFU spike threshold). We also set the magnitudes of the inhibitory connections to be weaker than the excitatory connections to prevent the IFUs from over-inhibition.

After selecting the SNs, stimulation sites, and clBCI weights, we started data collection, which began with 10–15 min of open-loop ICMS at 2–7 Hz (Poisson distributed pulses) to document each SN’s responses (open loop or ‘OL’ epoch) (figure 1(D)), and 5–10 min of recording spontaneous activity (‘Pre’ epoch). Table 1 lists the duration of each epoch for all experiments. During the OL epoch, stimulation was randomly interleaved across all channels to build a library of evoked spike and inhibitory responses for each SN. We then interfaced the selected SNs with the clBCI for 5–15 min to record the closed-loop dynamics (‘CL’ epoch). In two of the experiments for which time permitted, we collected 5 min of data following the closed-loop stimulation (‘post’ epoch). Altogether, we performed a total of 4 experiments, 3 in monkey J and 1 in monkey K.

**Table 1.** Durations of each experiment, as well as the firing and stimulation rates throughout each experimental condition.

Figure	OL (min)	Pre (min)	Pre (Hz)	CL (min)	CL (Hz)	CL stim rate (Hz)	Post (min)	Post (Hz)
**3**	0	5	R1, R2, R3 13.5, 21.9, 17.6	5	R1, R2, R3 14.4, 17.6, 15.7	S1, S2, S3 3.2, 3.6, 3.7	0	
**4**	10	5	R1, R2, R3, R4 20.5, 24.4, 18.1, 1.4	5	R1, R2, R3, R4 19.2, 17.3, 18.2, 5.8	S1, S2, S3, S4 5.3, 4.9, 5.7, 5.0	5	R1, R2, R3, R4 22.7, 23.6, 14.6, 1.5
**5**	15	10	R1, R2, R3, R4 51.4, 18.6, 8.7, 25.5	10	R1, R2, R3, R4 48.2, 21.1, 8.8, 17.6	S1, S2, S3, S4 7.3, 5.6, 3.1, 5.2	0	
**6**	10	5	R1, R2, R3 11.5, 19.9, 16.5	5	R1, R2, R3 14.8, 22.4, 14.0	S4, S5 2.3, 2.2	5	R1, R2, R3 14.3, 20.9, 11.0

### 2.5. Measuring feedback loops

clBCIs with recurrent connections between the IFUs and SNs could generate sustained patterns of evoked activity, henceforth referred to as feedback loops, whose characteristics depended on several factors including the number and connectivity between the SNs/IFUs, and the stimulation delays. To generate feedback loops, spontaneous spikes from the SNs could excite one or more IFUs, which then triggered ICMS. If an evoked spike was generated in one or more SNs, the evoked spikes could then together, or with other spontaneous spikes, excite more IFUs, which in turn could evoke more spikes, and so on.

To identify feedback loops from the CL SN spike data, we first identified each evoked spike, the corresponding IFU that triggered the ICMS, as well as both SN spikes that excited the IFU (as described above, by design, two SN spikes were sufficient to trigger an IFU spike). The three SN spikes (two triggering spikes and one evoked spike) were then grouped together to create so-called ‘elementary patterns’ (figure 1(E)). Elementary patterns with overlapping SN spikes were then grouped together to isolate the complete feedback loops (figure 1(E)). The size of each feedback loop was defined to be the total number of grouped SN spikes.

### 2.6. Simulating the closed-loop dynamics

To facilitate analysis of the closed-loop dynamics of the clBCIs, we developed a model in MATLAB to simulate the CL dynamics of the clBCI. The model consisted of two elements: (1) a Markov model that generated spike trains with the same statistics as recorded SNs, and (2) a stimulation-response model that simulated each SN’s response to ICMS.

**Figure 2.**
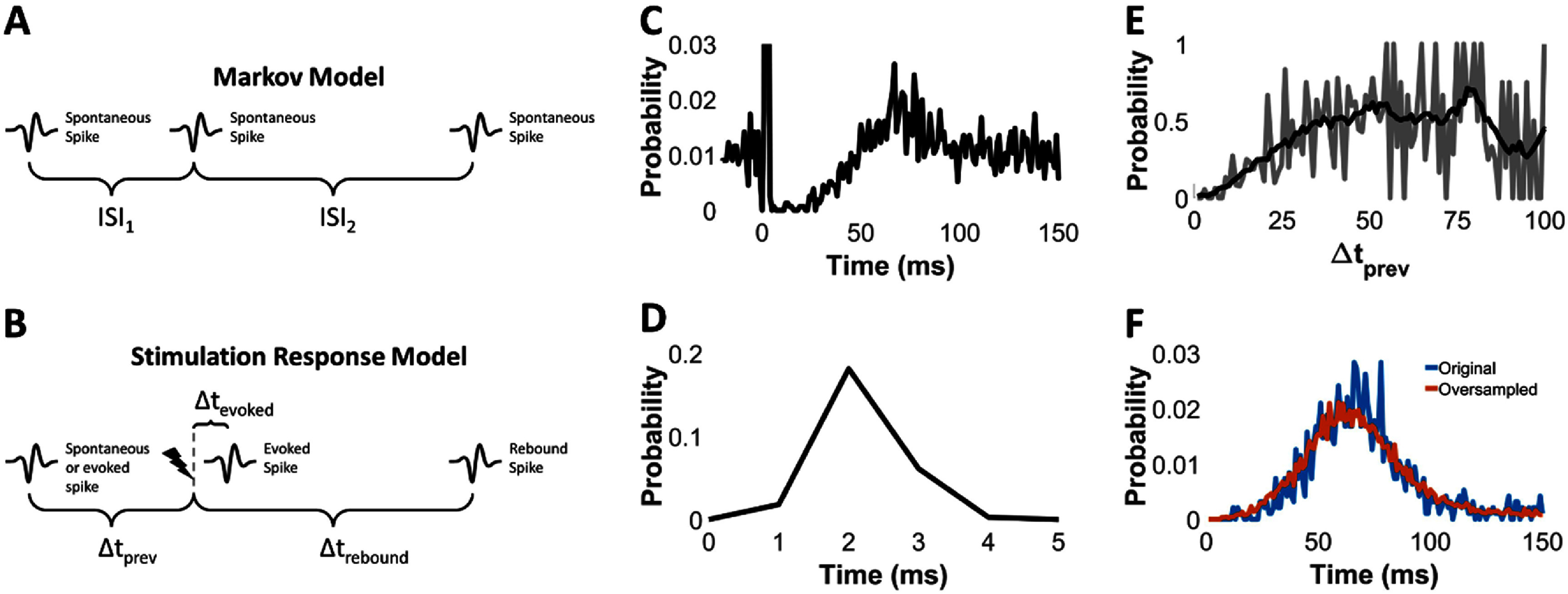
Simulating the closed-loop dynamics between SNs and IFUs. (A) Markov model to simulate spike trains from SNs. For every interspike interval, ISI_1_, from an SN, all possible interspike intervals, ISI_2_, that follow ISI_1_ are stored in an array. To generate a spike train, a seed is randomly selected from the ISI_1_’s, after which an ISI_2_ is randomly chosen from the array. This approximates sampling from the probability distribution P(ISI_2_ | ISI_1_) (B) Stimulation response model. The probability of evoking a spike after stimulation is dependent on the time interval Δt_prev_ between the previous spike time and stimulation onset and is measured from the data obtained in the OL epoch. Similarly, rebound spikes, which are observed at time intervals Δt_rebound_ after evoked spikes, are randomly sampled from the distribution of responses measured experimentally. (C) Example PSTH of R3 spike times locked to stimulation onset (S3 → R3 from figure 5) collected from the OL epoch. (D). PSTH of evoked spikes (S3 → R3 from figure 5). (E) Probability distribution of evoking a spike (S3 → R3 from figure 5) as a function of Δt_prev_. Grey curve shows unsmoothed distribution, black curve shows the distribution smoothed with a moving average over 10 ms. (F) Original and oversampled distributions of rebound spikes (S3 → R3 from figure 5) following stimulation.

The Markov model (figure 2(A)) was generated from the spike trains collected during the Pre epoch. To start, the interspike intervals of each recorded SN were calculated and rounded to the nearest millisecond. For each unique interspike interval, ISI_1_, every subsequent interspike interval, ISI_2_, was measured and stored in an array (supplementary figure 1(A)). To generate a simulated spike train, an ISI_1_ was randomly selected from the data to be the seed, after which an ISI_2_ was randomly selected according to the probability *P*(ISI_2_ | ISI_1_) as determined by the stored array. This process was repeated until a spike train of an arbitrary length was created.

The second component was the stimulation-response model, which was obtained from the OL epoch, and was comprised of the evoked spike and inhibitory responses (figure 2(B)). We first plotted the peristimulus time histograms for each SN and found the peaks corresponding to the evoked spikes (figures 2(C) and (D)). Any spike that occurred within this peak was counted as an evoked spike. Evoked doublets were infrequent, but we counted any second spike that occurred within 3 ms of the first evoked spike as part of the evoked response. As shown in our previous work [19], the probability of evoking a spike was dependent on the time interval between the last spike (rounded to the nearest millisecond) and the electrical stimulus (figure 2(E)). Due to the typically high rate of stimulation and high spike rate of the SNs during the OL epoch, the probability distribution of evoking spikes as a function of Δ*t*_prev_ had a low sampling density at larger values of Δ*t*_prev_ and a high variance. To address this, we smoothed these probability distributions using a 10 ms window moving average. Furthermore, for all values of Δ*t*_prev_ greater than 100 ms, we set the probability to be the average probability for evoking a spike in that SN. When an evoked spike was generated in our simulations, the timing of the evoked spike following the stimulus was randomly chosen from a distribution of timings Δ*t*_evoked_ obtained from the OL epoch data (supplementary figure 1(B)).

To model the inhibition, we extracted the collection of first non-evoked spikes (so-called ‘rebound spikes’) that occurred after stimulation (figure 2(F)). The distribution for rebound spikes was the same, independently of whether a spike was evoked or not [19]. To account for the short OL epochs, we then oversampled these distributions by taking 10 000 random samples from each distribution and adding uniformly distributed noise between −10 and 10 ms to each random sample, after which we combined these samples with the original distributions. Any rebound spikes in the modified distributions that occurred within the window of time for evoked spikes were discarded. Importantly, across our experiments, there were two SNs (clBCI shown in figure 4) in which the subsequent stimulus would frequently occur prior to the rebound spikes. We supplemented the Δ*t*_rebound_ for these two SNs with Gaussian distributions (supplementary figure 2). Finally, we incorporated stimulation artifacts into the model. Each artifact lasted 1 ms and obstructed every spike that occurred within that interval. If another stimulus (from another electrode) was set to occur within this 1 ms period, it was delayed until the end of the 1 ms.

## 3. Results

### 3.1. Recurrent connections between SNs and IFUs modulate SN spike dynamics

**Figure 3.**
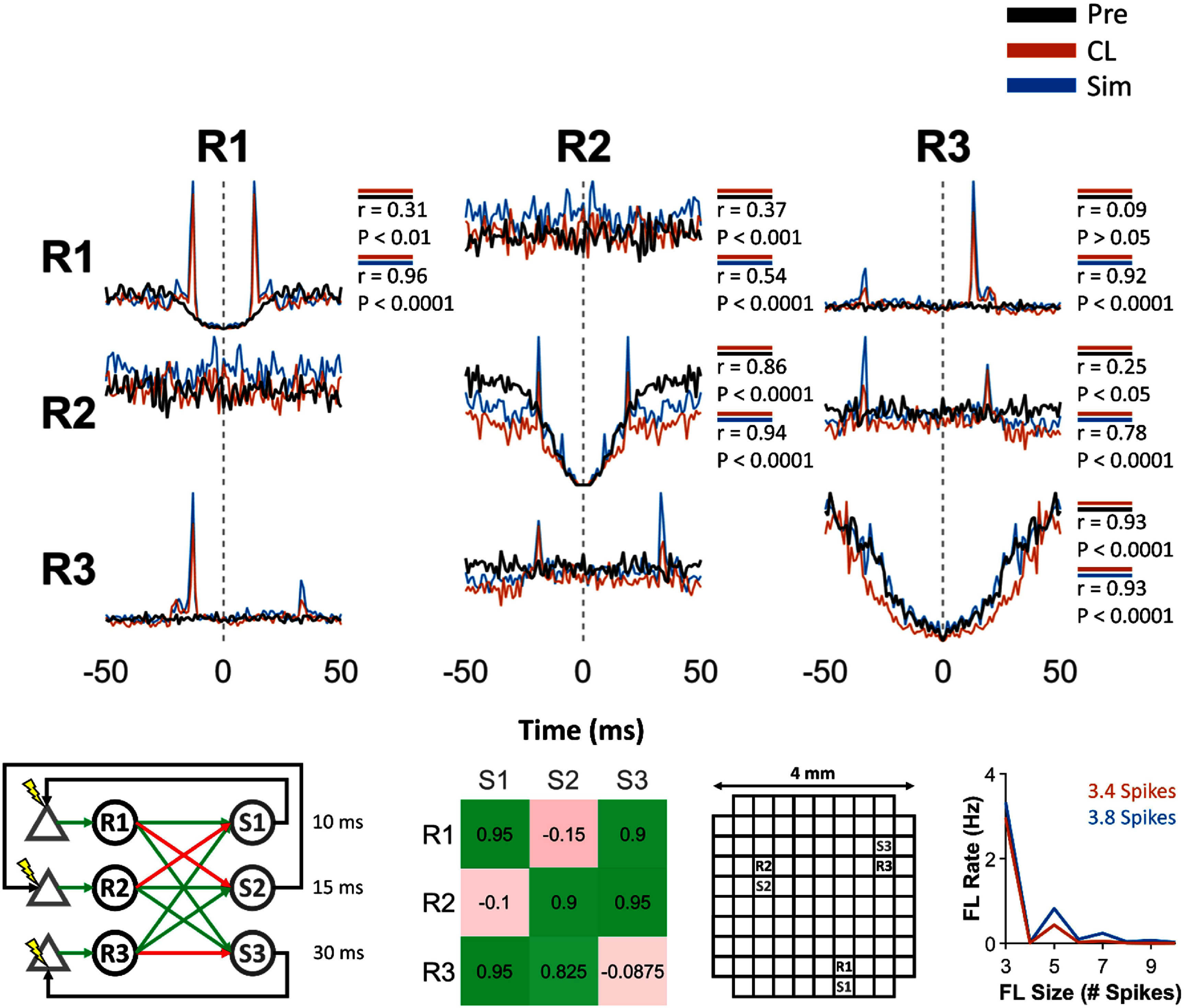
Spike dynamics during Pre epoch, CL epoch, and simulations for a clBCI with three SNs and three IFUs tested in monkey K. The top panel shows the correlograms across the Pre epoch (black), CL epoch (orange), and simulation of the CL dynamics (blue). The Pearson correlation coefficients between the correlograms during the Pre and CL epochs, as well as between the CL epoch and simulations, are shown for each condition. Across all correlograms, the Pearson correlation coefficients were consistently higher between the CL epoch and simulations than between the Pre and CL epochs, indicating that the simulated clBCI dynamics more closely captured the spike dynamics observed during closed-loop operation than those present during baseline activity. Note that the correlations between the correlograms are symmetric (i.e. R1 → R2 = R2 → R1), so we did not include the correlation coefficients for the correlograms along the lower diagonal. The bottom panels show the network architecture (green and red arrows show excitatory and inhibitory connections, respectively), IFU connection weights (defined as the fraction of the PSP amplitude to IFU activation threshold), locations of recording/stimulation channels on the Utah Array, and the rate of feedback loops of size N and their average sizes during the CL epoch and simulations.

**Figure 4.**
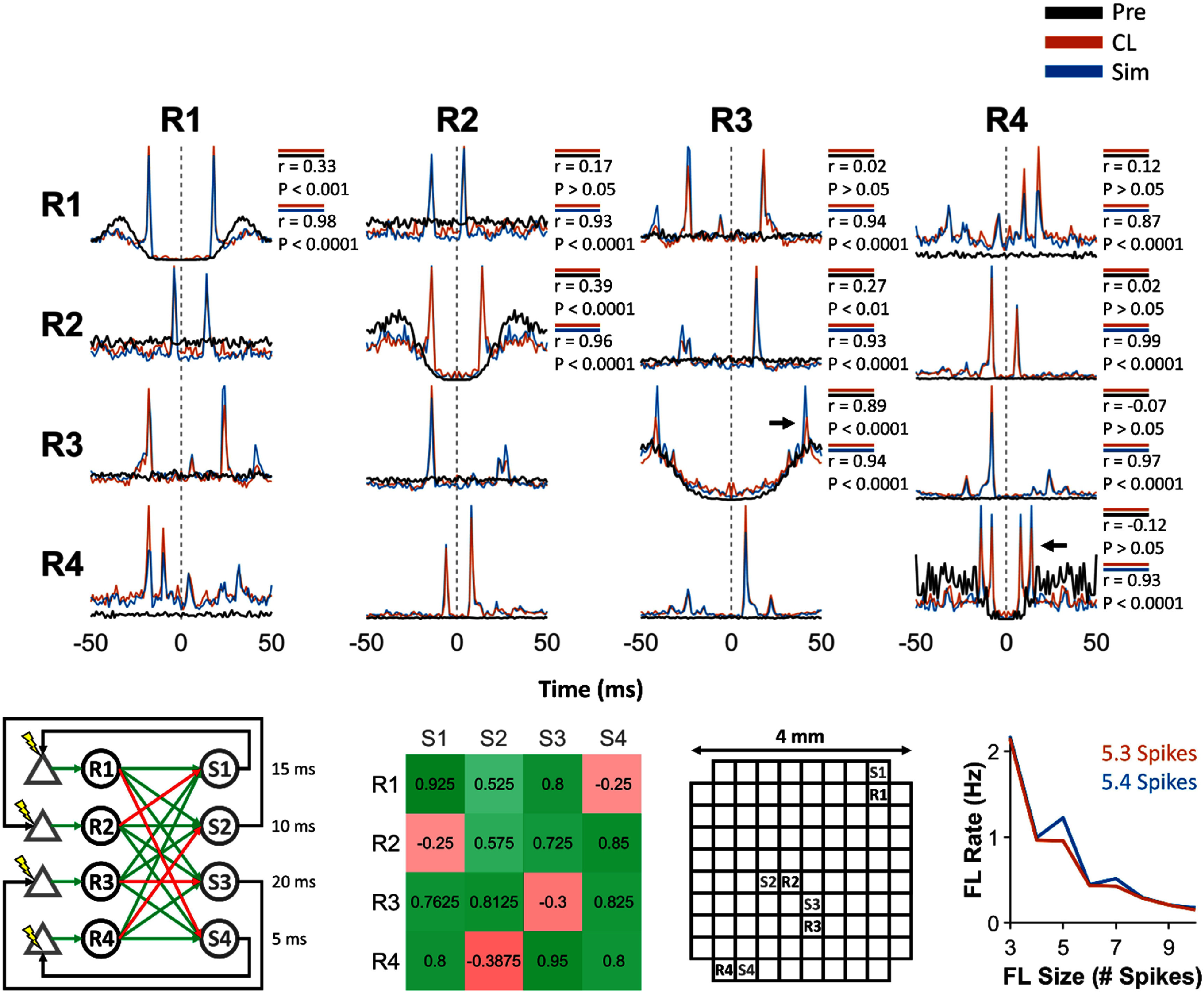
Same format as figure 3. Experiment performed in monkey J. The black arrows in the autocorrelograms of R3 and R4 show features in correlograms arising from feedback loops created by the clBCI.

**Figure 5.**
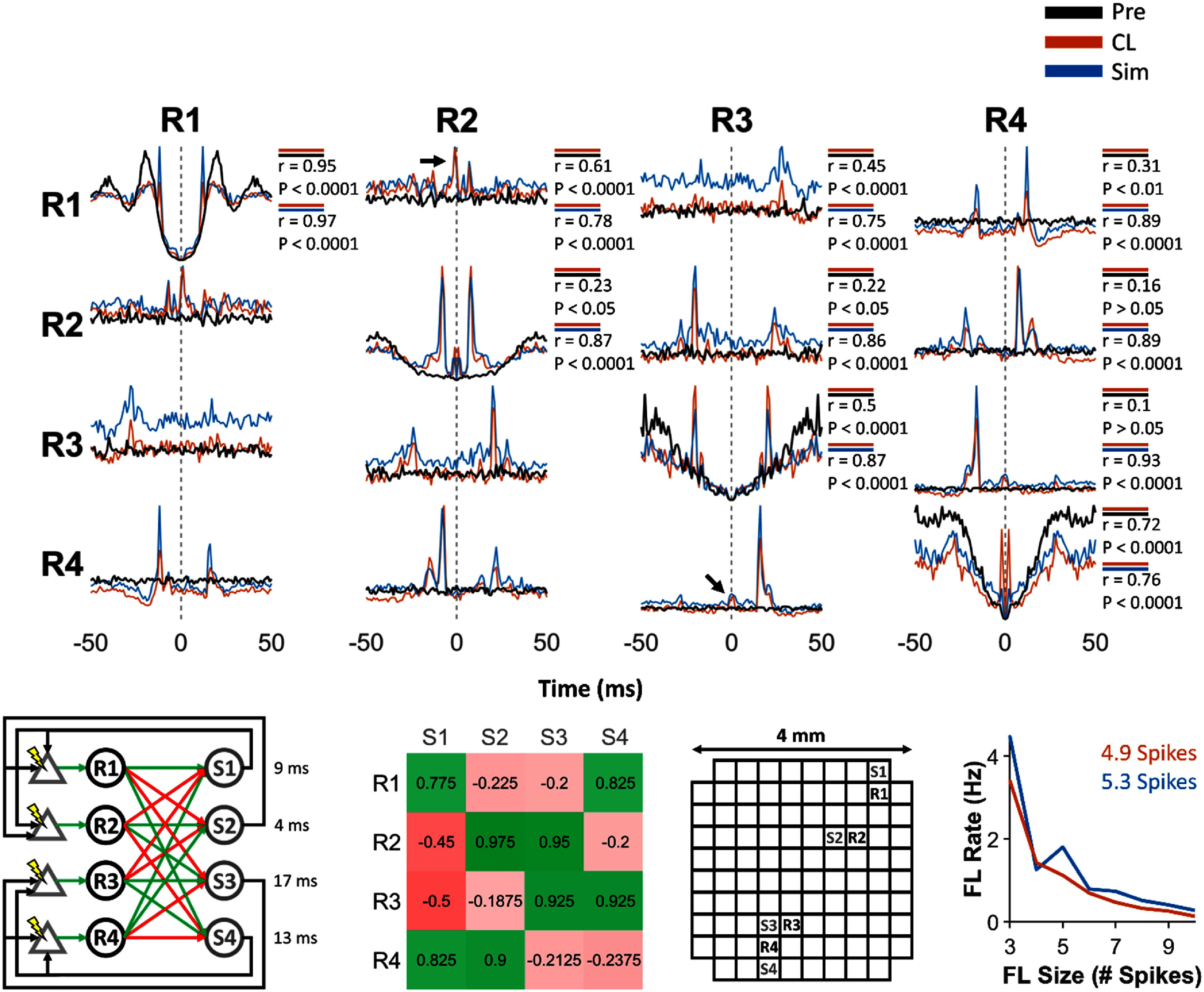
Same format as figure 3. Experiment performed in monkey J. The black arrows in the cross-correlograms of R1 and R2, as well as R3 and R4 show features in the correlograms arising from the ICMS exciting evoked spikes in both SNs with the same pulse.

**Figure 6.**
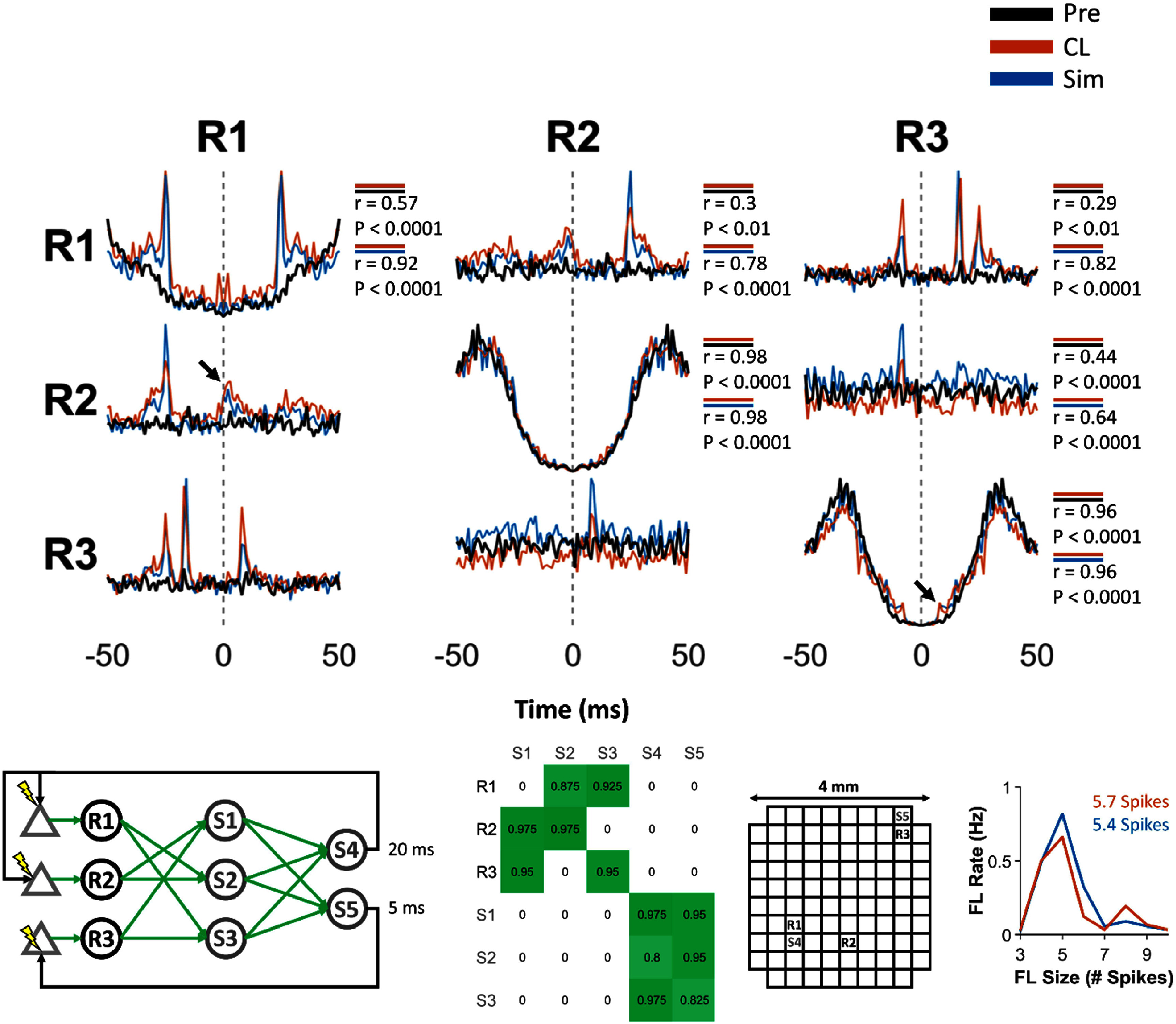
Same format as figure 3. Experiment performed in monkey J. Black arrow in cross-correlogram of R1 and R2 show co-excitation of the SNs from ICMS. Black arrow in autocorrelogram of R3 shows low amount of excitation due to the low probability of evoking a spike at small delays between the previous spike time and stimulation onset.

To establish a basic understanding of how recurrent connections between the SNs and artificial IFUs influenced the closed-loop dynamics of the SNs, we performed three experiments in which a small collection of SNs and IFUs were recurrently connected. The connections, weights, stimulation delays, and electrode positions are shown in figures 3–6 (bottom). Each IFU was manually selected to have a different unique combination of excitatory and inhibitory connections from the SNs. Throughout the experiments we implemented, the excitatory synaptic weights were tuned such that an IFU would only spike if it received two EPSPs within a narrow temporal window ranging from 1.1 to 12.6 ms, depending on the specific strengths of the connections. This temporal requirement effectively imposed a coincidence detection constraint on IFU activation, ensuring that only tightly coordinated presynaptic activity could drive postsynaptic spiking. In contrast, IPSPs served a suppressive role by reducing the membrane potential of the IFU, thereby counteracting incoming excitation. As a result, even when two SNs with excitatory connections to an IFU fired in close succession, this was often insufficient to elicit a spike if coincident or recent inhibitory input was also present (see methods section ‘Spiking neural network model’ for PSP modeling and implementation on the Neurochip3). Since each output IFU triggered a single pulse of ICMS, which in turn could evoke spikes and/or inhibit the activity of the SNs, the clBCIs created artificial connections between the SNs. We also found that in some of the clBCIs, the artificial connections between the SNs and IFUs could generate self-sustaining patterns of evoked activity (‘feedback loops’) in which the ICMS-evoked spikes could themselves excite the IFUs, thereby triggering more ICMS pulses to generate more evoked spikes (see methods section ‘Measuring Feedback Loops’ for more details).

We utilized spike train auto- and cross-correlograms of SNs to examine how clBCIs influenced SN spike dynamics relative to their baseline activity during the Pre epoch, which served as the no-stimulation control. Correlogram analyses are particularly well suited for this comparison because they directly reveal changes in spike timing relationships and connectivity patterns induced by closed-loop modulation. For this analysis, we first rounded the spike times of each SN to the nearest millisecond, after which we created binary vectors of each spike train. We then calculated the cross-correlations of these binary vectors (with the central point zeroed out for all autocorrelations). We found that the smaller clBCI, comprising just three SNs and three IFUs (figure 3, left), created simple artificial connections between the SNs, as seen in the auto- and cross-correlograms of the SNs’ spike trains (figure 3, top). The dashed grey lines in each column correspond to the spike times of each of the SNs indicated at the top, while the curves of each plot represent the probability of observing spikes from the SN listed on the left column within ±50 ms of those reference spike times. Peaks in these plots reflect increased temporal coupling between neurons indicative of the artificial connectivity induced by the clBCI. Note that in contrast to the Pre epoch (black), the CL epoch shows large peaks (orange) which correspond to the evoked spikes generated by the artificial connections between the SNs. Due to the small number of SNs and IFUs, we did not observe a substantial number of feedback loops (figure 3, bottom right). The average size of the feedback loops was 3.4 spikes, indicating that most loops consisted of only a brief sequence of activity—typically the initial trigger spikes followed by a single evoked spike. In such cases, the loop effectively represented a direct artificial connection between neurons rather than a truly self-sustained chain of activity.

Having established that a clBCI with three SNs and IFUs can modulate intrinsic spike activity, we next investigated how expanding the size of the clBCI would affect the closed-loop dynamics. As more SNs and IFUs are added to the clBCI, a greater number SNs will be artificially linked together to generate more complex closed-loop dynamics and feedback loops. To this end, we tested two clBCIs, each of which had four SNs and four IFUs with different connectivities. The first of these clBCIs, shown in figure 4, was an extension of the clBCI shown in figure 3, where each SN had three excitatory and one inhibitory connection to individual IFUs. Compared to the clBCI with three SNs and IFUs, we found that in addition to creating simple connections between the SNs, the clBCI generated consistent feedback loops that appeared in the autocorrelograms of R3 and R4 (black arrows). To illustrate how one such feedback loop was generated, when R1 and R3 spiked in close enough succession, both IFUs S1 and S2 would spike and trigger ICMS to evoke spikes in R1 and R2 at delays of 15 and 10 ms, respectively. These evoked spikes then excited S3, which triggered ICMS to evoke a spike In R3. Therefore, although R3 did not have a direct excitatory connection to S3 (the IFU that triggered ICMS to excite R3), it could indirectly spur its own excitation via a feedback loop.

The second clBCI was constructed in a similar manner to the configuration shown in figure 4, but with a key difference: each SN was connected to the IFUs via two excitatory and two inhibitory connections. This altered the balance of excitation and inhibition within the circuit and led to constraints on the resulting feedback loop dynamics. The increased proportion of inhibitory connections introduced two specific requirements for loop propagation. First, because each SN had only two excitatory connections, effective stimulation of the IFUs required either a single SN to fire two spikes in close succession or both SNs to fire in close succession, effectively raising the temporal precision needed for successful excitation. Second, the presence of more inhibitory projections introduced a gating effect in which the excitatory spikes could only evoke IFU activity if they occurred in a narrow temporal window during which the inhibitory SNs remained silent. Together, these conditions reduced the likelihood of IFU activation compared to the earlier configuration. To quantify the functional consequences of this more restrictive architecture, we calculated a stimulation efficiency ratio by dividing the total number of stimulation events by the total number of SN spikes across the session. A higher ratio indicates a greater proportion of spikes leading to stimulation, reflecting more efficient circuit excitation. As expected, the second clBCI configuration exhibited a reduced stimulation-to-spike ratio (0.23 stimuli/spike vs. 0.35 stimuli/spike), consistent with the suppressive influence of additional inhibitory inputs and the stricter temporal constraints required for effective activation.

In addition to feedback loops, we found other unexpected features in the CL dynamics, shown in the correlograms of figure 5. Although the stimulation sites were not initially designed to evoke spikes in multiple SNs, we found that S1 and S2 drove activity in both R1 and R2, while S3 and S4 excited both R3 and R4 (as shown in the clBCI architecture at the bottom left of figure 5). The resulting peaks in the correlograms, reflecting these multi-SN activations, are marked with black arrows in the correlograms. Altogether, these results demonstrate that the artificial connections between the SNs and artificial IFUs can create distinct closed-loop dynamics, as evidenced by the differences in correlograms and average feedback loop size/rate that depend on the clBCI architecture and the responses of the SNs to ICMS.

### 3.2. Hidden layers gate patterns of activation for the IFUs

Hidden layers in artificial rate- and spike-based neural networks increase their computational capabilities [25] and allow them to approximate any continuous function [26, 27]. With this in mind, we sought to document the effects of adding a hidden layer to an clBCI on the closed-loop dynamics. While two coincident excitatory inputs were sufficient to trigger IFU activation and ICMS pulses without a hidden layer, we hypothesized that adding a hidden layer of IFUs would introduce a gating mechanism—allowing only specific patterns of SN spikes, beyond simple pairwise coincidence, to drive ICMS.

Since the clBCIs were restricted to a total of eight SNs and IFUs in our setup, we implemented a network with three SNs, three artificial IFUs in a hidden layer, and two artificial IFUs in the output layer (figure 6). Because the IFUs were sparsely activated even with large weights, we did not add any inhibitory connections to the network. Although each SN was directly or indirectly connected to the output IFUs, the hidden layer introduced a gating effect on which patterns of SN spikes could excite the output IFUs. In contrast to the clBCIs with no hidden layers, where two coincident spikes from any SN could excite the output IFUs, the hidden layer altered this logic. In this configuration, a single SN could still trigger the output IFUs by firing twice within close succession, but spikes from two different SNs were insufficient to activate the output IFUs due to the lack of shared downstream connections. However, all three SNs firing in close succession was sufficient to drive the output IFUs. Another functional effect of this clBCI architecture was that the hidden layer extended the window of time for which the SNs could excite the IFUs in the output layer—R1, R2, and R3 could fire sequentially in any order over a period of time that outlasted the duration of the first EPSP and still trigger the IFUs in the output layer (supplementary figure 3). Finally, because R2 was activated by the same stimuli that activated R1, there was a broad peak in their cross-correlogram near 0 ms. However, due to the large stimulation delay and evoked spike latency for R2 (supplementary figure 1(B), bottom), its CL epoch autocorrelogram was very highly correlated (*r* = 0.98) with its Pre epoch autocorrelogram. These results suggest that it may be possible to create artificial correlations in cortical neural activity, while enabling individual SNs to retain their natural firing statistics.

### 3.3. The closed-loop dynamics can be predicted from open-loop measures

After performing the initial set of experiments *in vivo*, we sought to model the closed-loop dynamics between the SNs and IFUs to provide a tool to study their interactions in the absence of experimental constraints such as non-stationarities in the brain (supplementary figure 4), experimental run-time, and hardware limitations. The correlograms in figures 3–6 compare the simulated dynamics (blue) from each of the networks to the Pre (black) and CL (orange) dynamics. We found that the auto- and cross-correlograms generated by the simulation were consistently highly correlated with those from the CL epoch (*p* < 0.0001), whereas correlations between the Pre and CL epochs were either nonsignificant (*p* > 0.05) or markedly weaker. Notably, although the simulations were not conducted ahead of the closed-loop experiments, the model relied solely on spontaneous spike activity and open-loop responses from the same session (except for the one experiment where only spontaneous activity was collected prior to closed-loop testing) and still successfully predicted the closed-loop behavior.

It should be noted that for the clBCI in figure 3, we did not collect an OL epoch due to experimental time constraints. To simulate the CL dynamics for this clBCI, we extracted the stimulation responses from the CL portion of the experiment. While more rigorous testing will be required to understand the limits of being able to use the CL epoch to create the stimulation response model, the small network size and low stimulation rates likely contributed to it being an acceptable replacement.

### 3.4. Inhibition and stimulation delays impact feedback loop size

Having established that both the clBCI size and architecture influence the characteristics of the CL operation, we sought to document the influence of two other parameters—strength of inhibition and stimulation delays. For these analyses, we used our model to simulate the CL dynamics of the clBCIs shown in figures 4 and 6 with modified inhibitory strengths and stimulation delays, respectively. The EPSP magnitudes of the clBCIs in all simulations were unchanged from their original experimental values. The primary advantage of performing this analysis with the model is that similar networks can be tested free from the effects of non-stationarities in the brain, thus allowing for better comparisons. Each simulation was 5 min long. We used feedback loop size (the total combined number of spontaneous and evoked spikes in each feedback loop, see methods section ‘Measuring Feedback Loops’ for more details) as our primary metric to compare the effects of inhibition and stimulation delays since it serves as a single quantifiable measure of the closed-loop effects of the clBCI.

**Figure 7.**
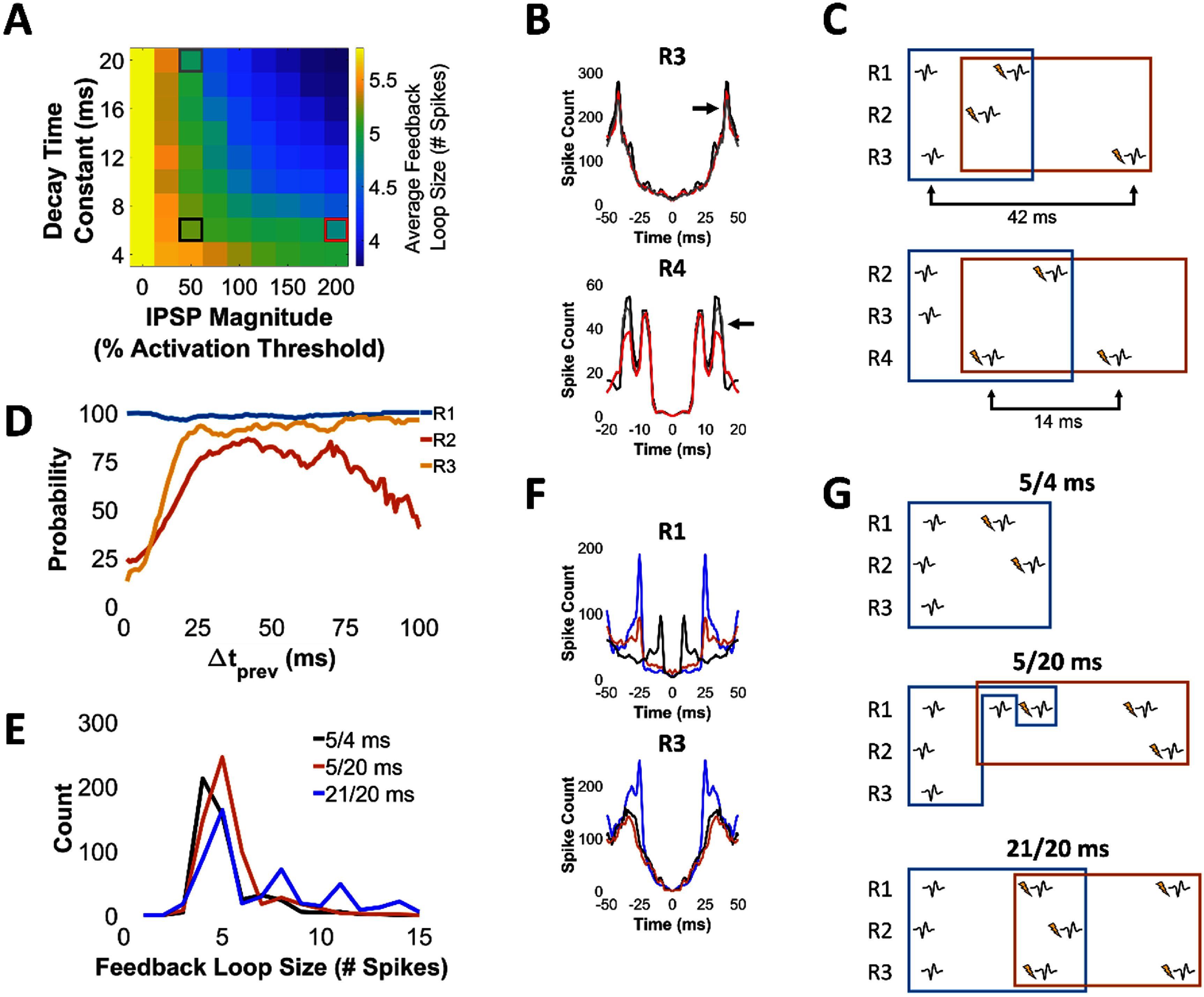
Effects of inhibition strength and stimulation delays on closed-loop spike dynamics. (A) Color plot of average feedback loop size showing monotonic effects of IPSP magnitude and decay time constant. Simulations were 5 min each and were performed using the clBCI in figure 4. Both variables significantly predicted the change in average feedback loop size in a linear regression model (*p* < 10^−16^). (B) Smoothed autocorrelograms (3 ms moving average) of R3 and R4 showing changes in feedback loops with increased IPSP magnitude (red) and increased IPSP decay time constant (gray). (C) Examples of how each corresponding feedback loop from (B) were generated. Times shown under arrows correspond to the peaks observed in (B) due to the feedback loops. (D) Probability of evoking a spike as a function of Δ*t*_prev_ for the clBCI shown in figure 6. (E) Number of feedback loop sizes between 0-15 spikes for the three different delay combinations used in the three simulations of the clBCI in figure 6 (see text for details). Feedback loops in 21/20 ms clBCI have the largest feedback loop sizes due to R2 and R3 having much larger probabilities of being evoked at larger values of Δ*t*_prev_. Feedback loop size distributions differed significantly across stimulation delay conditions (Kruskal–Wallis, *p* < 0.001), with all pairwise comparisons significant by post-hoc Wilcoxon rank-sum tests (all *p* < 0.001). (F) Autocorrelograms of R1 and R3 for each of the simulations in (E). (G) Examples of feedback loops for each of the three simulations in (E).

First, we explored how the strength of inhibition affected the CL dynamics by testing 81 different unique combinations of IPSP magnitudes (0%–200% of activation threshold of IFU units) and decay time constants (4–20 ms). Figure 7(A) shows a color plot of the average feedback loop size across all conditions, which shows a negative monotonic relationship between both IPSP magnitude and decay time constant with average feedback loop size. We used a linear regression model to characterize these relationships. Both variables significantly predicted the changes in feedback loop size (IPSP magnitude: *β* = − 0.002, *p* < 10^−16^; decay time: *β* = − 0.05, *p* < 10^−16^). The combined model accounted for *R*^2^ = 86.4% of the variance, confirming that both factors independently contribute to spike obstruction probability. While stronger levels of inhibition or more complex clBCI architectures may introduce nonlinear effects, these simulations were designed to explore basic circuit-level constraints under simplified conditions as a first step toward understanding how inhibition shapes closed-loop dynamics. Examples of how each of the feedback loops differed across three different conditions in the correlograms are shown in figures 7(B) and (C).

Next, we explored how the stimulation delays affected the feedback loop sizes with three simulations of different ICMS delays. We simulated three different delay combinations (5 4 ms^−1^, 5 20 ms^−1^, and 21 20 ms^−1^) based on the evoked spike probabilities of the SNs (figure 7(D)). Feedback loop size distributions differed significantly across stimulation delay conditions (Kruskal–Wallis, *p* < 0.001). Post-hoc Wilcoxon rank-sum tests confirmed that all pairs of delay conditions were significantly different (all *p* < 0.001), with longer stimulation delays resulting in broader distributions and a higher frequency of long-duration feedback loops. We found two separate mechanisms by which stimulation delay influenced feedback loop size. First, feedback loops were larger when stimulation was delivered at times with greater evoked spike probabilities (figures 7(E)–(G)). Second, feedback loop size was also dependent on the relative timing between ICMS pulses. When ICMS pulses from the two output IFUs were triggered closely in time, evoked spikes in the SNs were more likely to occur within a short window of time, thereby driving further IFU excitation (figures 7(E)–(G)). However, the relative timing of the two ICMS pulses had a smaller effect on feedback loop size than timing the ICMS to evoke spikes at greater probabilities. Despite timing the ICMS pulses closely together, feedback loop size was smaller in the clBCI that had stimulation delays with lower evoked spike probabilities compared to the clBCI that had much greater time separation of ICMS pulses, but with greater evoked spike probabilities (figures 7(E)–(G)).

### 3.5. Stimulation artifacts obscure spikes as a function of the number of SNs and IFUs

We sought to model the effects of stimulation artifacts on the performance of the clBCIs. For this analysis, we selected 13/14 of the SNs from our experiments (we excluded the SN R2 from figure 6, which was ostensibly evoked polysynaptically, due to its high variability in evoked spike latency). As with the *in vivo* experiments, any two stimuli that were set to occur within the same interval were staggered in time. For each of the sets of simulations, we tested clBCIs with 4–10 SNs/IFUs each in size increments of 2. Each IFU triggered stimulation at a randomly assigned delay of n*4, for *n* = 1,2…10, to keep each of the delays at least 4 ms apart to minimize the amount of staggered stimulation artifacts. To isolate the effects of increased network size, rather than the total number of synaptic connections, we restricted each IFU to only having 4 random presynaptic connections from the SNs. Furthermore, no inhibitory connections or hidden layers were used to simplify the analysis and gain a basic understanding of the relationships between network size, weights, and the number stimulus-obstructed spikes. For each of these clBCIs, we varied the magnitude of the weights between 50-87.5% of the activation threshold of the IFUs in increments of 12.5%. We repeated these simulations 10 times each with random connectivities, SNs, and stimulation delays to obtain an average number of stimulus-obstructed spikes.

In the first set of simulations, the stimuli did not evoke any spikes or inhibition but simply obstructed the detection of spikes for 1 ms following each stimulus, which served as a baseline measure. In the second set, each stimulus could evoke a spike and/or inhibit one of the SNs, while obstructing the detection of the spikes from the remaining SNs for 1 ms.

**Figure 8.**
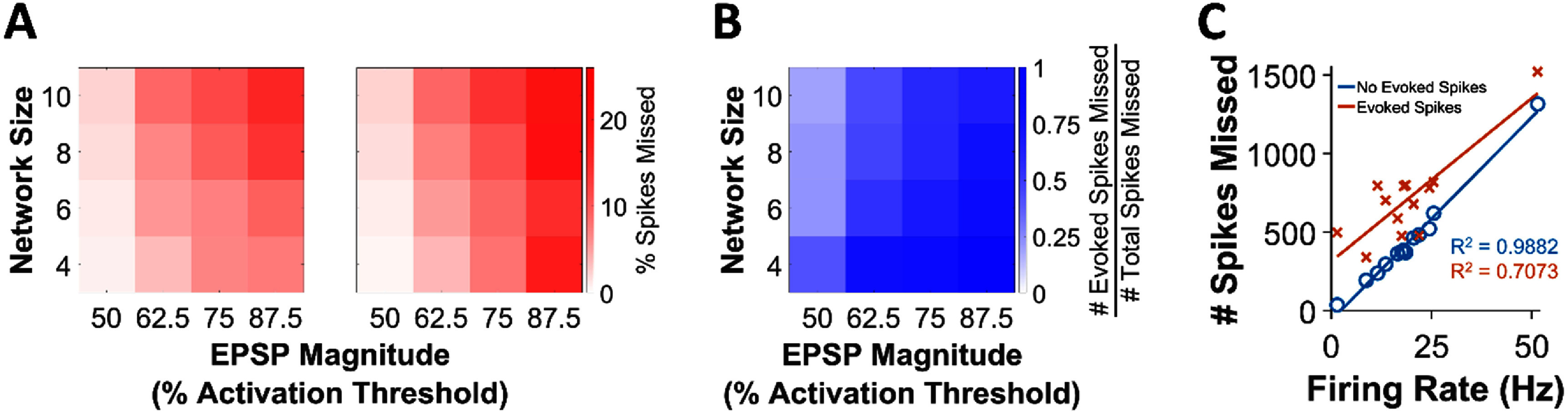
Quantifying the effects of stimulation artifacts. (A) Percentage of missed spikes as a function of EPSP magnitude (measured as % of activation threshold of IFUs) and network size (number of SNs and IFUs) for the simulations with no evoked spikes (left) and with evoked spikes (right). Linear regression revealed that both connection strength and network size significantly predicted the fraction of missed spikes in both simulation conditions (all *p* < 10^−3^), with *R*^2^ = 93.1% without evoked spikes and *R*^2^ = 96.1% with evoked spikes. (B) Ratio of missed spikes that were evoked spikes over all spikes (evoked and spontaneous spikes) for the simulations with evoked spikes. (C) Relationship between the firing rate of the SNs and the number of spikes that were missed due to stimulation artifacts. An ANCOVA-style analysis further showed that evoked spikes increased the baseline missed spike rate (*p* = 4.9 × 10^−4^), but did not significantly alter the relationship between firing rate and spike loss (*p* = 0.23). The slightly lower *R*^2^ in the evoked spike condition at high connection strengths reflects increased variability in evoked spike probabilities and inhibition duration, which disrupted the otherwise monotonic relationship between network parameters and spike obstruction.

Figure 8(A) shows the results of each set of simulations. For the simulations in which stimulation did not evoke any spikes or inhibit activity, the percentage of missed spikes had a clear monotonic relationship with the magnitude of the weights and network size. We used a linear regression model to evaluate the effect of connection strength and network size on spike obstruction. Both variables significantly predicted the fraction of missed spikes (weights: *β* = 0.0026, *p* < 10^−7^; network size: *β* = 0.0084, *p* < 10^−4^). The combined model accounted for *R*^2^ = 93.1% of the variance, confirming that both factors independently contribute to spike obstruction probability. When we included evoked spikes into the simulations, we again found that both variables significantly predicted the fraction of missed spikes (weights: *β* = 0.0042, *p* < 10^−9^; network size: *β* = 0.0067, *p* < 10^−3^). The combined model accounted for *R*^2^ = 96.1% of the variance. Interestingly, when evoked spikes were included, this relationship became less clear when the magnitude of the weights increased to 87.5% of the IFU activation threshold. This occurred because the outputs of the IFUs were more synchronized in smaller clBCIs due to greater number of overlapping connections. The synchronous IFU spikes in turn synchronized the subsequent evoked spikes which created large feedback loops in the SNs. The higher rate of stimulation outputs prevented spontaneous spikes from occurring, leading to a greater fraction of the obstructed spikes being evoked (figure 8(B)). Although clBCIs with more SNs and IFUs did not generate as many feedback loops, the larger network size increased the number of stimulation outputs which ultimately caused the clBCIs to obstruct a similar number of spikes.

Finally, we combined each set of ten simulations to measure the average number of spikes missed for each SN as a function of its spontaneous firing rate (figure 8(C)). We compared the linear relationship between neuronal firing rate and the percentage of missed spikes across two simulation conditions (with and without evoked spikes) using an ANCOVA-style linear regression with an interaction term. This model revealed a significant difference in intercepts between conditions (evoked vs. non-evoked: *p* = 4.9 × 10^−4^), indicating that the inclusion of evoked spikes led to a higher baseline missed spike rate. However, the interaction between firing rate and condition was not significant (*p* = 0.23), suggesting that the slope relating firing rate to missed spikes did not differ significantly between the two conditions.

When the stimulation did not evoke any spikes, there was a strong linear relationship between the firing rate of the SN and the number of spikes that were obstructed (*R*^2^ = 0.99). When evoked spikes were included, we found that the total number of missed spikes was greater, although the relationship between firing rate and number of evoked spikes had more variance (*R*^2^ = 0.7) due to the varying evoked spike probabilities and durations of inhibition. These results suggest that the incorporation of the ICMS-evoked responses strongly influence the extent to which the stimulation artifacts themselves degrade the performance of clBCI.

## 4. Discussion

Our study provides a first set of results from pilot experiments and simulations investigating how an artificial spiking neural network implemented on a clBCI interfaced with the cortex of awake NHPs shapes the closed-loop dynamics of SNs. We found that the combination of two simple models—a Markov model to simulate spike trains, and a stimulation-response model to simulate the responses of SNs to ICMS—was capable of accurately reproducing the closed-loop dynamics from open-loop measures. Of particular interest was that the recurrent nature of the artificial networks generated feedback loops whose characteristics were dependent on both the endogenous characteristics of the SNs as well as properties of the clBCI such as connectivity and stimulation delays between artificial neurons. The uniqueness of the feedback loops based on the pattern of SN spikes could potentially enable the clBCIs to generate input-specific, artificial short-term memories in the SNs, similarly to other kinds of artificial recurrent neural networks [28, 29].

Because ICMS typically generates both excitatory and inhibitory responses in the SNs, it may also be possible to train the clBCIs to control the firing rates of the SNs in addition to their precise relative timings of their action potentials. Although both excitatory and inhibitory responses can occur following each pulse of ICMS, the probability of evoking a spike in a SN for any given stimulus is a function of the delay between its previous spike time and stimulation onset [19]. Thus, by appropriately timing the stimulation, the relative amount of excitation and inhibition could be controlled. Due to the diversity in responses to ICMS, the degree to which the firing rate of any given SN can be increased or decreased is a function of several characteristics, including its spontaneous firing statistics, evoked spike probability, and average duration of inhibition. One important consideration, however, is that the ability of the clBCI to shape SN spike dynamics also depends on minimizing the number of missed spikes caused by stimulation artifacts. For this reason, an ideal training algorithm should aim to minimize the number of missed spikes by appropriately timing each stimulus pulse to simultaneously maximize the amount of desired excitation or inhibition, as well as stagger evoked spikes between the stimulation artifacts. Nevertheless, any training algorithm will need to carefully balance the tradeoffs between creating the desired patterns of activity and increasing the number of artifacts. With this in mind, there are numerous efforts underway to develop systems capable of subtracting the artifacts out of the recordings, including blind source separation [30], and adaptive filtering techniques [31], which will mitigate this limitation. Although real-time artifact subtraction remains technically challenging, particularly when amplifier saturation occurs, a combined approach of timing ICMS to maximize evoked spike probability with predictive signal recovery algorithms may further reduce artifact-induced signal loss.

Another important consideration for using this clBCI for shaping neuronal activity is its spatial resolution of neural recruitment. ICMS has consistently been shown to sparsely excite neurons proximal to the electrode tip as well as those more distal [32, 33], which we also found in this study. Although this presents a challenge to large-scale implementations of our clBCI, there are strategies that could mitigate this limitation. Through juxtacellular positioning of the electrodes, the amount of current delivered could be significantly reduced to increase the spatial resolution of ICMS. Furthermore, the stimulation could be selectively delivered during specific contexts, such as when specific circuit patterns of activity are detected to minimize disruption of endogenous activity. Importantly, our model was able to predict the effects of ICMS on neurons recorded from channels distant from the stimulation site (figures 5 and 6), demonstrating that even with distal neuronal activation, the closed-loop impact on neural activity is predictable.

The spiking IFUs utilized a simple temporal coding scheme of detecting coincident inputs, but more sophisticated SNN models such as the Izhikevich model could be used to increase the complexity of the temporal code by simulating the biophysical properties of various cell types [34, 35]. Future studies can utilize our model of closed-loop dynamics to more thoroughly probe how clBCIs can be optimally constructed to generate unique short-term memories or sustained neural activity for different inputs. Our computational model also provides a means of developing and testing training algorithms *in silico* rather than *in vivo*, avoiding challenges such as non-stationarities in firing rates and responses to ICMS (supplementary figure 4). One challenge, however, is the time required to build a response library using open-loop ICMS. In our experiments, we found that 5 min of 2–7 Hz stimulation was sufficient to characterize responses in up to 4 SNs. While this rate of 4 SNs per 5 min may seem impractical for mapping responses across hundreds or thousands of neurons, prior studies have shown that ICMS at the lower currents used here evokes more spatially restricted activation with increasing distance from the electrode [32, 33]. Therefore, a larger library of neuronal responses could likely be acquired by stimulating multiple cortical sites over a broad set of regions simultaneously. To train the clBCI, gradient-decent-based methods such as backpropagation have been proposed to train traditional firing-rate-based artificial neural networks that interface with the brain [17, 18], but these methods are not applicable to training the weights, stimulation delays and stimulation amplitudes in the clBCIs. Therefore, a reinforcement or evolutionary learning algorithm that could be used to efficiently explore the parameter space may be a better approach to developing future training strategies [36, 37].

While our study did not involve training the neural network to optimize a task or goal, our results pave the way for such an effort by both documenting and modeling the closed-loop dynamics. One future consideration would be how the brain may adapt to long-term closed-loop ICMS, for instance, through homeostatic mechanisms [38]. In two of our experiments in which we recorded a Post epoch, we did not observe any changes in activity following closed-loop stimulation (supplementary figure 5). Whether the brain adapts to extended periods of closed-loop stimulation was not investigated due to the challenges in reliably recording extracellular action potentials from the same SNs continuously for several days. It will be essential to explore this question further to develop an understanding of how the clBCIs can be designed to co-adapt to any such changes.

A long-term goal of this work is to develop a clBCI capable of precisely manipulating damaged neural circuits in a context-specific manner to restore function. Although implementing a clBCI that interfaces with thousands to millions of neurons presents substantial technical challenges, there are clinically meaningful applications even at smaller scales. For instance, some completely paralyzed patients already use BCIs to communicate and interact with their environments [39]. A scaled-up version of our clBCI—capable of learning and co-adapting with the brain—could assist patients in discovering the optimal patterns of SN activity that produce successful outcomes. By monitoring which combinations of natural and evoked activity yield reward, the clBCI’s training algorithm could iteratively adjust internal parameters such as synaptic connectivity, stimulation timing, and amplitude to accelerate the discovery of effective neural patterns. Toward this goal, the ability to model the closed-loop dynamics of clBCIs offers a critical framework for designing and refining such adaptive training strategies.

## 5. Conclusion

This study demonstrates that single cortical neurons in awake NHPs can be interfaced with artificial spiking neural networks *in vivo* to manipulate their activity. We show that the altered closed-loop activity of the cortical neurons can be modeled from open-loop measures of their endogenous spike activity and responses to stimulation. The model provided a means of testing different parameters of the spiking neural network such as inhibition strength and stimulation delay and could be used to design novel training algorithms that shape the activity of the cortical neurons in a context-specific manner. Finally, we show how the ability of the clBCI to function degrades as a function of the number of interfaced biological neurons and IFUs due to increases in stimulation artifacts that prevent the detection of spikes and discuss how this limitation may be overcome. Our results pave the way for the development of clBCIs that can repair neurological damage by artificially rewiring the connectivities of biological neurons.

## Data Availability

The data that support the findings of this study are openly available at the following URL/DOI: https://figshare.com/articles/dataset/BBCI_Raw_Data_and_Processing_Code/28012994.
